# Biomarkers for predicting response to long‐term high dose aspirin therapy in aspirin‐exacerbated respiratory disease

**DOI:** 10.1002/clt2.12048

**Published:** 2021-08-13

**Authors:** Lucyna Mastalerz, Katarzyna E. Tyrak

**Affiliations:** ^1^ 2nd Department of Internal Medicine Jagiellonian University Medical College Cracow Poland

**Keywords:** aspirin therapy after aspirin desensitization, aspirin‐exacerbated respiratory disease, responders and non‐responders

## Abstract

**Introduction:**

Aspirin‐exacerbated respiratory disease (AERD) is a phenotype of asthma characterized by eosinophilic inflammation in the airways, mast cell activation, cysteinyl leukotriene overproduction, and acute respiratory reactions on exposure to cyclooxygenase‐1 inhibitors. Aspirin desensitization followed by daily high‐dose aspirin therapy is a safe and effective treatment option for the majority of patients with AERD. However, there is still some percentage of the population who do not derive benefits from daily aspirin use.

**Methods:**

Based on the current literature, the biomarkers, which might predict aspirin treatment outcomes in AERD patients, were evaluated.

**Results and conclusions:**

Patients with severe symptoms of chronic rhinosinusitis, type 2 asthma based on blood eosinophilia, non‐neutrophilic inflammatory phenotype based on sputum cells, as well as high plasma level of 15‐hydroxyeicosatetraenoic acid (15‐HETE) are potentially good responders to long term high‐dose aspirin therapy. Additionally, high expression of the hydroxyprostaglandin dehydrogenase gene, *HPGD* encoding prostaglandin‐degrading enzyme 15‐hydroxyprostaglandin dehydrogenase (15‐PGDH) and low expression of the proteoglycan 2 gene, *PRG2* encoding constituent of the eosinophil granule in sputum cells might serve as a predictor of good response to aspirin therapy. Variations in the expression of cysteinyl leukotriene receptor 1 in the airways could additionally influence the response to long‐term aspirin therapy. Arachidonic acid metabolites levels via the 5‐lipoxygenase as well as via the cyclooxygenase pathways in induced sputum supernatant do not change during high dose long‐term aspirin therapy and do not influence outcomes of aspirin treatment.

## 1 INTRODUCTION

Aspirin‐exacerbated respiratory disease (AERD) is a phenotype of asthma characterized by eosinophilic inflammation in the airways, mast cell activation, cysteinyl leukotrienes (cysLTs) overproduction, and acute respiratory reactions on exposure to cyclooxygenase‐1 (COX‐1) inhibitors.^1^, ^2^ Aspirin desensitization followed by daily high‐dose aspirin therapy can be offered as a therapeutic option to most patients with AERD.^3^ ^,^ ^4^ ^,^ ^5^ ^,^ ^6^ ^,^ ^7^ ^,^ ^8^ ^,^ ^9^ This is supported by studies reporting that 82% to 87% of these patients experience improvement on long‐term aspirin treatment.^3^, ^4^ However, there is still a percentage of individuals who do not derive benefits from such therapy, while predictive factors of response to aspirin remain poorly understood. Therefore, the assessment of eligibility for and prediction of response to aspirin therapy continue to be clinically relevant issues. A critical evaluation of the current evidence on predictors of response to aspirin therapy might result in recommendations formulated in line with the key principles of personalized medicine, which would facilitate clinical management. Laidlaw et al. recommended aspirin therapy at a dose of 650 mg twice daily for an initial period of 8 weeks, with a subsequent dose titration to 325 mg twice daily to assess whether the lower dose can maintain symptom control.^6^ From clinical perspective, the identification of responders is of outmost importance as aspirin has side effects and its chronic use might lead to, among others, bleeding, gastrointestinal symptoms and liver or renal failure. On the other hand, it is very effective in patients after myocardial infarction or suffering from severe back or joint pains.

In this paper, various clinical, inflammatory, molecular and genetic characteristics of AERD patients were assessed to determine which factors, if any, might influence or predict good response to long‐term high dose aspirin therapy.

## 2 CLINICAL PREDICTORS FOR GOOD RESPONSE TO ASPIRIN THERAPY

Significant differences in response to aspirin therapy were observe among different ethnic groups. Among patients who did not respond to aspirin treatment and even experienced the worsening of respiratory symptoms, 75% were African‐Americans and 25% were Latinos. In contrast, none of the white, non‐Latino patients worsened on aspirin.^10^ Data concerning differences in aspirin therapy outcomes by gender are inconsistent. In one study female sex was proposed to be positive prognostic factor, as only one woman (4% of females) did not benefit from treatment.^4^ There is also a study showing no difference in treatment outcomes by gender.^10^ Responders to aspirin were also characterized by severe nasal symptoms based on 22‐item Sino‐Nasal Outcome Test (SNOT‐22) and a longer period from sinus surgery to aspirin desensitization than non‐responders. On the other hand, in a study by Jerschow et al. patients with more severe sinus disease based on computed tomography scans were less likely to complete the desensitization protocol.^10^ It was suggested that aspirin challenge/desensitization should be proposed to patients shortly after sinus surgery when their aspirin‐induced hypersensitivity reactions become less severe.^11^, ^12^

On the basis of the above, it can be concluded that data concerning demographic and clinical characteristics of AERD patients and their potential association with response to aspirin treatment are rather contradictory. Further studies are needed to make conclusions which demographic and clinical data, if any, could serve as predictors for response to aspirin treatment.

## 3 SEVERE TYPE 2 ASTHMA BASED ON PERIPHERAL EOSINOPHILIA AS THE GREEN LIGHT FOR ASPIRIN THERAPY

Type 2 (T2) inflammatory asthma is associated with eosinophil and mast cell activation as well as type 2 cytokine release for example IL‐5.^13^, ^14^ T2 inflammatory profile of severe asthma was defined as blood eosinophil count of 150 cells/μl or higher or (and) IS eosinophil percentage of 2% or higher based on the 2020 Global Initiative for Asthma (GINA) update.^15^ According to Sze et al., T2 asthma was classified as eosinophil percentage of 3% or higher and neutrophil percentage lower than 64%^16^ in induced sputum. Higher blood eosinophil count and lower neutrophil percentage in sputum were potentially associated with good response to aspirin treatment in AERD patients with severe asthma defined as 1) increase in prebronchodilator FEV_1_ by at least 100 ml; 2) increase in the Asthma Control Questionnaire‐ 5 (ACQ‐5) score by at least 0.5 points; and 3) reduction in the SNOT‐22 score by at least 9 points.^4^ Although peripheral blood eosinophil count may not always accurately represent the cellular state of the asthmatic airways. AERD patients with type 2 (T2) asthma are considered eligible for long‐term high‐dose aspirin treatment independent of atopy status. Immunoglobulin E (IgE) is a prominent biomarker for early onset asthma, but its levels are also often elevated in late‐onset nonallergic asthma. The pattern of IgE expression in the latter is mostly polyclonal and frequently associated with blood eosinophilia.^17^, ^18^ It seems that the innate immune response is the key driver in this asthma phenotype. The release of interleukins IL‐25 and IL‐33 as well as thymic stromal lymphopoietin (TSLP) from the respiratory epithelium, which activates T2 innate lymphoid cells (ILC2s) via its soluble receptor ST2, lead to a release of T2 cytokines (IL‐4, IL‐5, and IL‐13) from ILC2s and T2‐helper (Th2) cells, with subsequent mast cell degranulation, massive local B‐cell activation with IgE formation, and finally, eosinophil attraction.^19^ ^,^ ^20^ ^,^ ^21^ Interleukin 5 is potently released by ILC2s and Th2 cells; therefore, blood and tissue eosinophilia can serve as evidence of inflammation driven by both cell types.^22^, ^23^ During long‐term high‐dose aspirin therapy, this process was suppressed locally (in the airways) but not globally (in the blood) in patients with severe asthma and aspirin hypersensitivity who responded to aspirin therapy.^4^ The threshold value for blood eosinophil count that best differentiates AERD responders from nonresponders to high‐dose aspirin should be determined with consideration of asthma severity and potential confounding local effects of corticosteroids and other immune modulators.^4^, ^24^ Inhaled corticosteroids, which are the first‐line therapy for asthma, can reduce eosinophil count, as demonstrated by Cowan et al.^25^

Paradoxically, high‐dose aspirin therapy was reported to increase systemic markers of T2 inflammation, such as peripheral blood eosinophilia,^4^, ^9^ mast cell activation assessed by plasma tryptase levels,^9^ and urinary leukotriene E_4_ production in AERD patients^4^, ^9^ independently of response to aspirin, despite reducing bronchial and nasal symptoms. No significant changes with no trends across timepoints in T2 cytokine profile were observed in either blood^9^ or induced sputum supernatant in patients with AERD on daily aspirin therapy.^4^ The local response to aspirin was different than the global one. There was a significant decrease in sputum eosinophil percentage in responders, while no changes were observed in nonresponders. Moreover, in responders, blood eosinophil count remained stable on aspirin.^4^ The question arises whether it is possible that aspirin might act as a mediator favoring T‐cell plasticity. It is known that each T‐cell type can repolarize toward the other when treated with the opposing cytokine.^26^, ^27^ The blood eosinophil count is an established parameter for identifying patients with severe asthma^28^ who are likely to respond to long‐term aspirin therapy targeting T2 cytokines.^4^, ^29^ Low neutrophil count in sputum may also be an important predictor of good response to aspirin.^4^ Patients with an inflammatory neutrophilic phenotype based on induced sputum are not eligible for aspirin treatment. The threshold value that best differentiated responders was 48.5% or lower for sputum neutrophils. Therefore, the identification of a non‐T2 profile is essential as it rather excludes aspirin treatment in AERD patients.

### 3.1 The balance of molecular phenotypes – a prerequisite for aspirin therapy?

**FIGURE 1:**
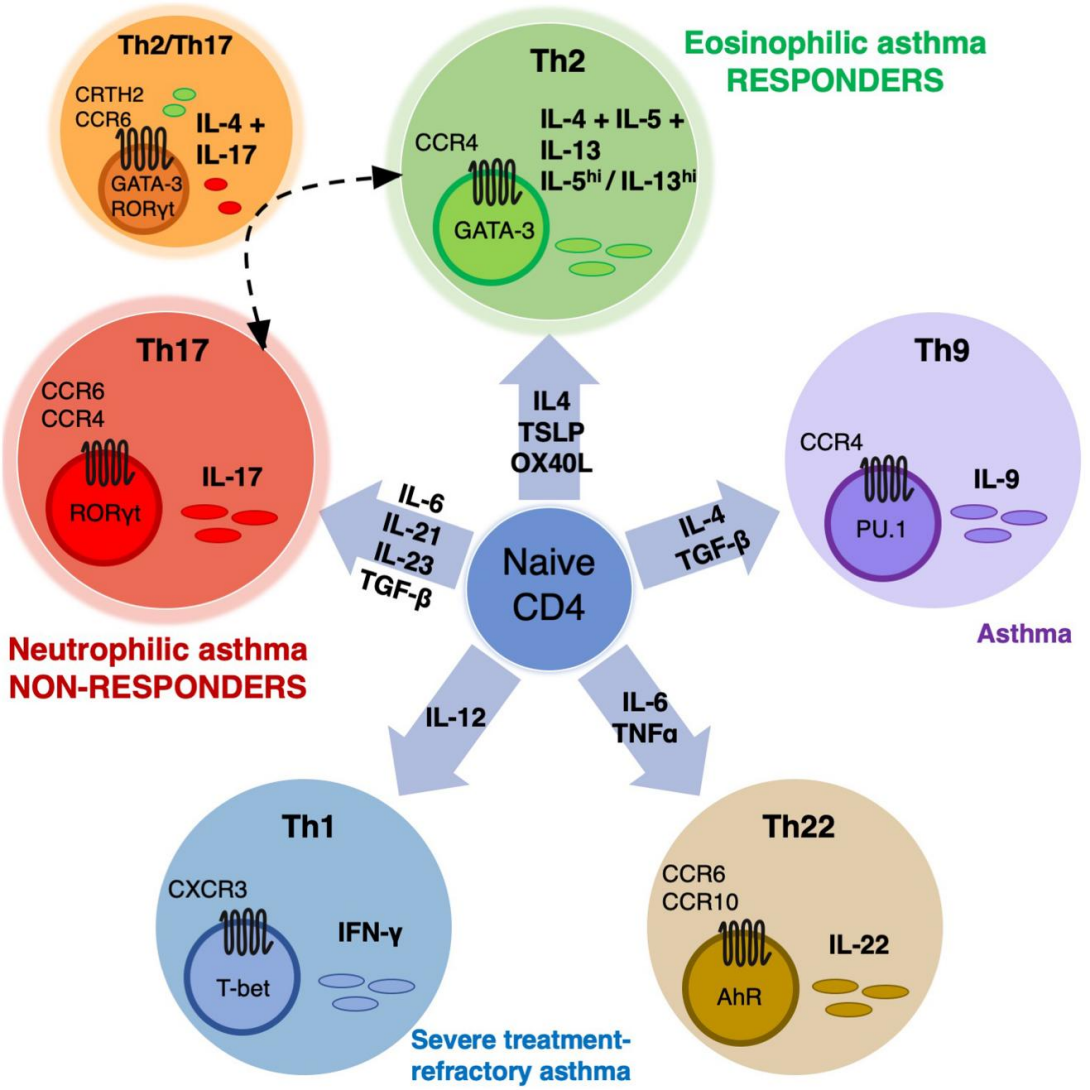
Naïve CD4 cells differentiate into distinct Th subsets in response to different cytokines. Aspirin‐exacerbated respiratory disease is a heterogeneous disease with a spectrum of subphenotypes according to the type of inflammation present in a given patient. Aspirin‐exacerbated respiratory disease patients with type 2 asthma based on blood eosinophilia will respond to high‐dose aspirin treatment, while patients with the neutrophilic inflammatory phenotype based on sputum will not respond to aspirin therapy. A minority of aspirin‐exacerbated respiratory disease patients have neutrophilic phenotype associated with Th17 cytokines. The Th2 and Th17 pathways regulate one another‐if the balance tilts towards Th17, then patients with the neutrophilic phenotype will not respond to aspirin treatment. AhR‐aryl hydrocarbon receptor, CCR4‐ C‐C Motif Chemokine Receptor 4, CCR6‐ C‐C Motif Chemokine Receptor 6, CCR‐10‐ C‐C Motif Chemokine Receptor 10, CRTH2‐ prostaglandin D2 receptor 2, GATA‐3‐ GATA Binding Protein 3, PU.1‐ transcription factor PU.1, RORγt‐ Retineic‐acid‐receptor‐related orphan nuclear receptor gamma, TGF‐*β*‐ Transforming growth factor beta, TNF‐α‐ tumor necrosis factor α

Aspirin‐exacerbated respiratory disease is a heterogeneous disease with a spectrum of subphenotypes based on lower and upper airway inflammation.^30^, ^31^ Type 2–related cytokines (from Th2 and ILC2) play a central role in regulating inflammatory processes in patients with AERD.^32^ Wenzel et al. proposed the classification of asthma into the Th2/T2 and non‐Th2/T2 phenotypes, according to the type of inflammation present in a given patient.^33^ Based on induced sputum, a minority of AERD patients have neutrophilic phenotype (non‐Th2/T2) associated with Th17 cytokines.^34^ Indeed, the Th2 and Th17 pathways are mutually exclusive and regulate one another, favoring the development of Th2 immune responses in patients with chronic rhinosinusitis with nasal polyposis (CRSwNP).^35^ It can be assumed that if the balance of these two pathways tilts towards Th17, then patients with the neutrophilic phenotype will not respond to aspirin treatment (Figure 1).

## 4 GENETIC PREDICTORS

### 4.1 Expression of the HPGD gene in sputum cells

Regular aspirin use was associated with a lower incidence of asthma exacerbations and improvement of nasal symptoms in the majority of patients with severe asthma classified as responders. Those who responded to treatment were characterized by high baseline expression of the hydroxyprostaglandin dehydrogenase gene, HPGD, in sputum cells.^4^ This suggests that high HPGD gene expression in sputum cells may serve as a biomarker predicting benefits from aspirin therapy. The HPGD gene encodes prostaglandin‐degrading enzyme 15‐hydroxyprostaglandin dehydrogenase (15‐PGDH), which is a functional antagonist of cyclooxygenase‐2 (COX‐2).^36^ Notably, COX‐2 downregulation has been reported in the nasal polyps (NPs) of patients with asthma and aspirin hypersensitivity.^37^ Arachidonic acid is preferentially metabolized by COX‐2 to anti‐inflammatory prostanoids such as prostacyclin and prostaglandin E_2_ (PGE_2_).^38^ It was speculated that the COX‐2/HPGD system functions as a complex network potentially regulating response to high‐dose aspirin therapy in AERD patients by stabilizing COX‐2 expression.^4^ These data suggest that in the course of aspirin treatment, COX‐2 may in fact play a protective role by producing PGE_2_, which stabilizes mast cell activation and inhibits ILC2s.^9^, ^39^, ^40^ Stable production of PGE_2_ in the airways might be one of the several factors contributing to well‐controlled asthma and improved nasal symptoms on aspirin.^4^ This hypothesis was supported by clinical studies showing that exogenous PGE_2_ prevents bronchospasm induced by aspirin in patients with AERD^41^ ^,^ ^42^ An increased COX‐2 expression induced by aspirin was also documented in some cell types in humans.^43^ Additionally, activated mast cells due to the depletion of COX‐1–derived PGE_2_ on aspirin expressed significantly higher levels of mRNA encoding COX‐2 than of that encoding COX‐1.^44^

### 4.2 PRG2 gene expression in sputum cells

The protein encoded by proteoglycan 2, pro eosinophil major basic protein gene, PRG2, is the predominant constituent of the crystalline core of the eosinophil granule.^45^ The granule crystalloid core is composed of major basic protein with well‐known biochemical and functional properties.^46^ This protein might be involved in immune hypersensitivity reactions by being directly implicated in epithelial cell damage and bronchospasm in patients with asthma.^45^ Indeed, a higher baseline expression of the PRG2 gene in sputum cells of AERD patients, indicating potential greater activity of eosinophils, determined the lack of response to long‐term aspirin treatment. Thus, it was supposed that the activity of eosinophils might be more important than their quantity in bronchial mucosa as far as response to high‐dose aspirin therapy is concerned. A low PRG2 gene expression level in sputum cells may be a predictor of a stronger benefit from aspirin therapy in AERD patients.^4^

### 4.3 CYSLTR1 gene expression in sputum cells

The main cellular sources of cysLTs include mast cells, eosinophils, and platelet‐adherent granulocyte activation.^2^, ^47^ Most of the proinflammatory actions of the cysLTs are mediated by their binding to the CysLT1 receptor, encoded by CYSLTR1 gene.^48^ Indeed, AERD patients are characterized by a selective increase in the expression of CysLT1 receptor on nasal submucosal inflammatory cells compared with asthma patients with chronic rhinosinusitis with nasal polyposis who tolerate aspirin well.^49^ Desensitization followed by intranasal lysine aspirin therapy was associated with a decrease in the numbers of inflammatory cells expressing CysLT1 receptors in nasal mucosa. As this leads to the lower activation of key proinflammatory cells by cysLTs, such as eosinophils and monocytes, the authors speculated that this mechanism might contribute to the therapeutic benefit of aspirin.^49^ Different levels of CysLT1 receptor expression in a target organ may contribute to the heterogeneity of responses to long‐term aspirin therapy. Stable expression of the CysLT1 receptor in sputum cells during aspirin therapy may lead to the decreased recruitment of eosinophils, further diminishing the inflammatory reaction by reduced secretion of their granule contents.^50^ ^,^ ^51^

### 4.4 Interleukin‐4/STAT‐6 inhibition

The differentiation of Th2 cells from naïve CD4 T cells proceed typically in the presence of IL‐4 in the local cytokine milieu and is associated with the dimerization of signal transducer and activator of transcription‐6 (STAT6).^52^ Similarly, stimulation of B cell by IL‐4 and/or IL‐13 leads to phosphorylation, dimerization and nuclear migration of STAT6, which induces gene transcription and promotes B cell isotype class switching.^53^ Long‐term treatment with aspirin was associated with the suppression of sputum IL‐4 release.^29^ Indeed, the inhibition of STAT6 by aspirin has been reported in CD4 T cells^54^ as a primary source of IL‐4, thus providing explanation for the observed reduction^29^ or at least stable levels of IL‐4 in sputum during aspirin treatment.^4^ The reduction of local IL‐4 levels mediates tolerance reactions to aspirin during long‐term aspirin therapy in patients with hypersensitivity. Further studies on STAT6 gene expression in sputum and nasal cells in AERD patients who respond to aspirin therapy are warranted.

## 5 ARACHIDONIC ACID METABOLITE'S PREDICTOR OF RESPONSE TO ASPIRIN THERAPY

Plasma 15‐hydroxyeicosatetraenoic acid (15‐HETE) predicts treatment outcomes in AERD. Higher 15‐HETE level at baseline and its strong correlation with eosinophilia at 4 weeks of aspirin therapy is characteristic for patients who benefited from aspirin therapy. This indicates a possible role of baseline 15‐HETE as a predictor of aspirin treatment outcomes in AERD patients.^10^ 15‐HETE is derived from arachidonic acid via the 15‐lipoxygenase (15‐LO) pathway and eosinophils, bronchial epithelium as well as nasal epithelial cells^55^ have a high capacity to produce this eicosanoid. In patients with eosinophilic inflammatory phenotype of severe asthma compared to patients without airway eosinophilia, higher levels of 15‐HETE were observed in bronchoalveolar lavage fluid.^55^ Notably, 15‐HETE is associated with eosinophilic inflammation in AERD patients. Arachidonic acid metabolites levels via the 5‐LO as well as via the COX pathways in induced sputum supernatant do not change during high dose, long‐term aspirin therapy and do not influence outcomes of aspirin treatment.^4^ It is unknown whether 15‐HETE is affected by aspirin desensitization and whether its levels change during aspirin treatment in AERD.

**FIGURE 2:**
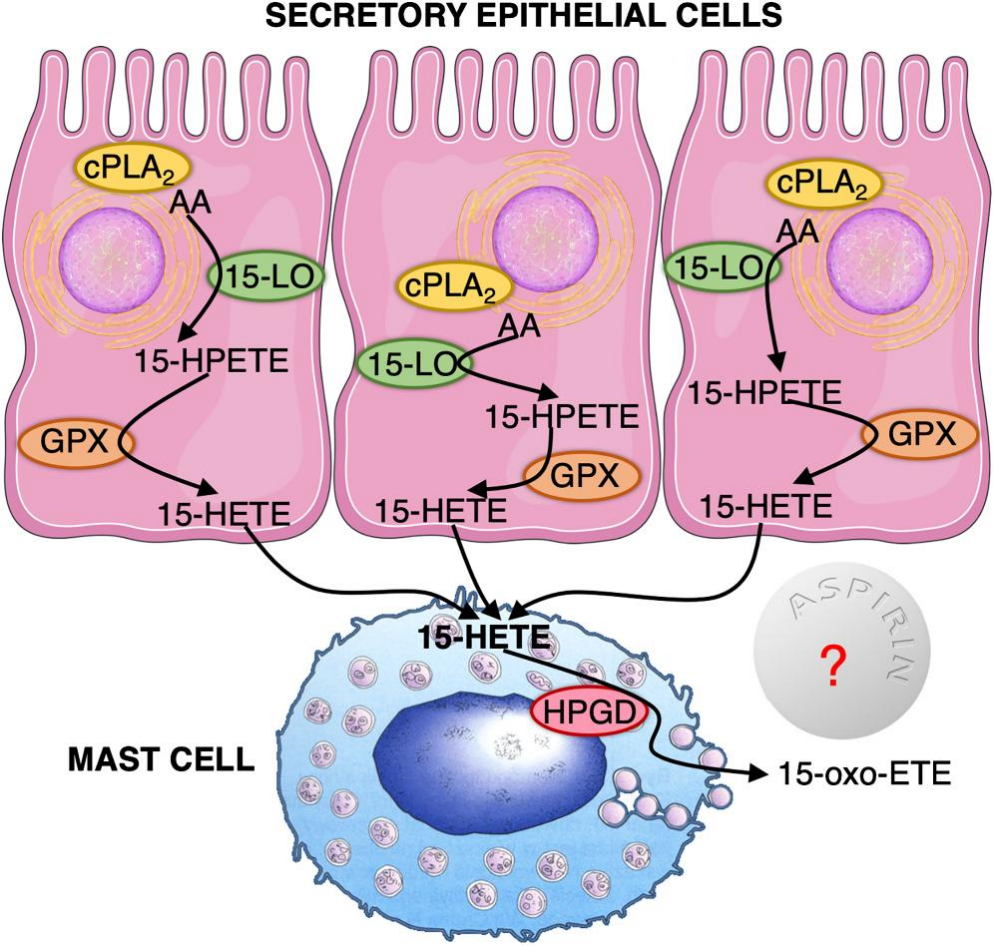
Adapted from Dwyer DF, *J Allergy Clin Immunol. 2021;147:501‐503*. Epithelial and mast cell interactions lead to 15‐Oxo‐eicosatetraenoic acid (15‐Oxo‐ETE) synthesis. Responders to aspirin treatment are characterized by high baseline expression of the hydroxyprostaglandin dehydrogenase gene, HPGD, in sputum cells. HPGD encodes prostaglandin‐degrading enzyme 15‐hydroxyprostaglandin dehydrogenase (15‐PGDH), which is required for 15‐Oxo‐ETE synthesis

In the context of high sputum *HPGD* expression, encoding hydroxyprostaglandin dehydrogenase, in AERD patients who respond to aspirin, some interesting results have been published.^55^ Notably, The *ALOX15* encoding 15‐LO was significantly elevated in NPs of patients with AERD compared to NPs of patients with CRSwNP or healthy controls. *ALOX15* was predominantly expressed by epithelial cells. Expression levels significantly correlated with clinical severity of asthma and radiographical sinus disease. The level of 15‐oxo‐eicosatetraenoic acid (15‐Oxo‐ETE), a downstream product of 15‐LO, was markedly higher in tissue of nasal polyposis of AERD patients compared to CRwNP and inferior turbinate tissue from controls. Interestingly, hydroxyprostaglandin dehydrogenase is required for 15‐Oxo‐ETE synthesis, was predominantly expressed in mast cells and localized near 15‐LO epithelium in NPs from patients with AERD. Indeed, we need a study, which would investigate whether 15‐Oxo‐ETE is affected by aspirin desensitization and whether its levels change during aspirin treatment in AERD. It was concluded that epithelial and mast cell interactions, leading to the synthesis of 15‐Oxo‐ETE, may contribute to the dysregulation of arachidonic acid metabolism via the 15‐LO pathway in AERD patients.^55^ This indicates a possible role of baseline 15‐Oxo‐ETE as predictor of aspirin treatment outcomes in AERD patients (Figure 2).

## 6 CONCLUSIONS

**FIGURE 3:**
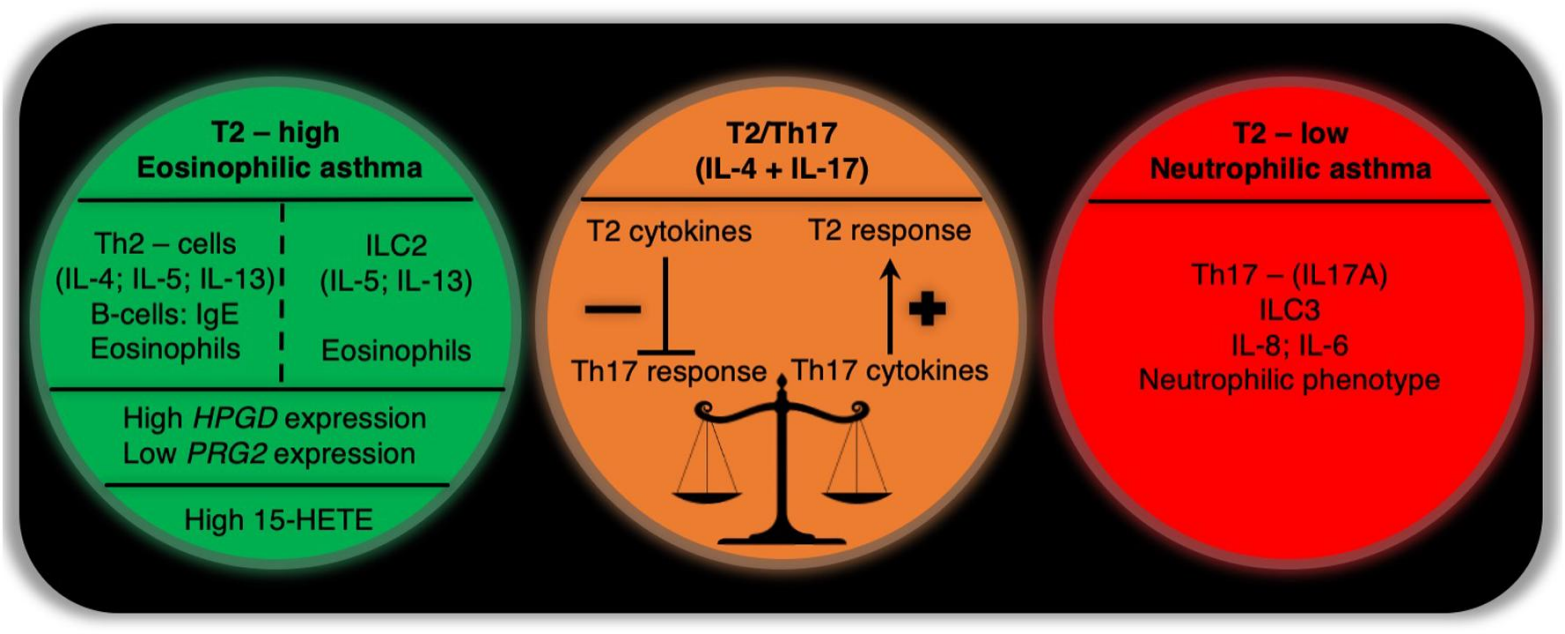
Predictors of response and the lack of response to long‐term aspirin therapy offers the key principles of personalized medicine in aspirin‐exacerbated respiratory disease (AERD) patients with severe asthma. HPGD‐hydroxyprostaglandin dehydrogenase; PRG2‐proteoglycan 2, pro eosinophil major basic protein; 15‐HETE‐ 15‐hydroxyeicosatetraenoic acid

AERD patients with severe nasal symptoms, type 2 asthma independent of atopy status, and with non‐neutrophilic phenotype based on sputum cells will respond to high‐dose aspirin treatment. High sputum cell expression of *HPGD* encoding prostaglandin degrading enzyme, and low expression of *PRG2* encoding constituent of the eosinophil granule might serve as a predictor of good response to aspirin treatment. The expression of genes associated with arachidonate acid pathways encoding prostaglandin‐endoperoxide synthase 1, prostaglandin‐endoperoxide synthase 2, arachidonate 5‐lipooxygenase, leukotriene C_4_ synthase, 12‐lipooxygenase, 15‐lipooxygenase as well as for prostaglandin E receptor 2, prostaglandin E receptor 4, prostaglandin E_2_ receptor 2, CysLT1 and CysLT2 receptors in sputum cells will not be helpful in predicting response to long‐term aspirin therapy in AERD. Notably, the evaluation of most eicosanoids (prostaglandin D2, prostaglandin E2, 8‐iso‐prostaglandin E2, tetranor‐prostaglandin D‐M, tetranor‐prostaglandin E‐M, leukotriene B4, cysLTs) except for 15‐HETE in induced sputum supernatant cannot predict response to long‐term aspirin therapy in AERD.^4^ High plasma 15‐HETE level at baseline is characteristic for AERD patients who benefited from aspirin therapy.^10^ Variations in the expression of CysLT1 receptor in the airways could also influence the response to long‐term aspirin therapy.^49–51^ Clinical, inflammatory, molecular and genetic predictors of response to long‐term high dose aspirin therapy in aspirin‐exacerbated respiratory disease are presented in Figure 3.

## AUTHOR CONTRIBUTIONS

Lucyna Mastalerz and Katarzyna E. Tyrak wrote the first draft of the manuscript, incorporated all subsequent changes, as well as coordinated the final version and submission of the manuscript.

## CONFLICTS OF INTEREST

The authors declare no conflict of interest.
